# Insight into the nitrogen accumulation in urban center river from functional genes and bacterial community

**DOI:** 10.1371/journal.pone.0238531

**Published:** 2020-09-02

**Authors:** Lei Yu, ShuLei Liu, LiJuan Jiang, XiaoLin Wang, Lin Xiao

**Affiliations:** School of the Environment, State Key Laboratory for Pollution Control and Resource Reuse (SKL-PCRR), Nanjing University, Nanjing, China; Chinese Academy of Sciences, CHINA

## Abstract

Along with urbanization, the intensified nitrogen pollution in urban rivers and the form of black-odor rivers has become one of the biggest concerns. Better understanding of the nitrogen transformations and microbial mechanisms occurring within urban rivers could help to manage their water quality. In this study, pollution characteristics, potential nitrogen removal rate, composition and function of bacterial community, and abundance of functional genes associated with nitrogen transformation were comparatively investigated in a typical urban river (FC) and a suburban river (LH). Compared with LH, FC was characterized by higher content of nutrients, lower potential nitrogen removal rate and lower abundance of functional genes associated with nitrogen transformation in both overlying water and sediment, especially in summer. Sediment dissolved organic matter characterized by excitation−emission matrix (EEM) showed that FC was more severely polluted by high nitrogen organic matter. Our results revealed that anammox was the main nitrogen removal pathway in both rivers and potential nitrogen removal rates decreased significantly in summer. Bacterial community analysis showed that the benthic communities were more severely influenced by the pollutant than aquatic ones in both rivers. Furthermore, the FC benthic community was dominated by anaerobic respiring, fermentative, sulfate reduction bacteria. Quantitatively, the denitrification rate showed a significant positive correlation with the abundance of denitrification genes, whilst the anammox rate was significantly negatively correlated with bacterial diversity. Meanwhile, NH_4_^+^-N had a significant negative correlation to both denitrification and anammox in sediment. Taken together, the results indicated that the increased nitrogen pollutants in an urban river altered nitrogen removal pathways and bacterial communities, which could in turn exacerbate the nitrogen pollution to this river.

## Introduction

Fast growth of population and urbanization cause urban areas to produce more and more pollution. Although improved wastewater collection and treatment can efficiently control point source pollution, nonpoint source pollution, such as surface runoff caused by rainfall events, as well as endogenous release of the sediment pollutants persist to increase the levels of pollution in urban rivers [1–4]. Usually, urban rivers are characterized by high levels of nutrient inputs associated with human activities [1, 5]. More seriously, many rivers heavily polluted by organic matter, nitrogen, phosphorus, and heavy metals often resulted in excessive oxygen consumption and production of blackening and stinking pollutants, which finally caused rivers to become black and odor smell, especially in summer [6–8]. Nowadays, urban water quality degradation had been observed in developed and developing countries [6, 9]. Stress to local water resources had become one of the biggest concerns associated with urbanization [1, 10]. Therefore, improving urban river water quality, especially elimination of black-odor phenomenon in rivers, is an urgent need for sustainable city development.

High loading of nitrogen (N) is considered as one of the main causes of black-odor phenomenon in rivers [1, 2, 6, 8]. Self-purification function of riverine ecosystems can removal N pollutants through microbial driven processes of ammonification, nitrification, denitrification and anoxic ammonium oxidation (anammox) [11]. However, N removal processes are influenced by variation of environmental factors, contaminants and microbial communities [11–15]. Water quality also deeply influences river ecosystem structure, food-web integrity, as well as ecosystem functions and services [16]. Previous studies have demonstrated that flow velocity and contaminants in urban rivers affected sediment oxygen demand, and the structure and integrity of eukaryotic and bacterial communities [14, 17, 18]. The affected microbial communities in turn disrupted microbial driven self-purification processes, which might aggravate the accumulation of pollution and water quality degradation [8, 19]. For example, effluent inputs and seasonal changes determined the abundance and distribution of nitrifiers, and subsequent transformation of NH_4_^+^-N to nitrate in urban rivers [20]. Till now, many studies about the deterioration of water quality in urban rivers focused on the influent pollution, as well as mechanisms underlying the phenomenon of black and odor in urban rivers [14, 15, 20, 21]. It is pressing to investigate the characteristics of N accumulation and removal in urban rivers and underlying microbial mechanisms to take steps to improve water quality and protect urban aquatic ecosystems.

Wuxi city, adjacent to Lake Taihu, has witnessed degradation of urban river water quality along with fast urbanization. FengChan river (FC) is located in the city center of Wuxi and the FC subwatershed is dominated by urban land cover. LiangHong river (LH) is situated in a wetland protection area within suburb of Wuxi city and is isolated from direct urban impact. FC and LH provide an opportunity to distinguish the influence of the characteristics of urban and suburban pollution into the river, as well as their effects on microbial community structure and self-purification function in rivers. In this study, we compared the biogeochemical properties, bacterial community composition, functional gene abundance, and potential N removal processes in FC and LH. And we discussed the factors that influence the potential N removal processes. The results would help to systematically understand the influence of urbanization on river N pollution and removal at the level of community, functional gene and bacterial denitrification capacity. The results will provide scientific clues to manage and restore urban river water quality.

## Materials and methods

### Study sites and sampling

In this study, water and sediment samples were collected from an urban river (31°36 N, 120°18 E; named FC in this study) located in the city center of Wuxi and a river located in the LiangHong wetland protection area (31°30 N, 120°31 E; named LH in this study). The two rivers have similar temperature and climate environment. Water and surface sediment samples were collected in April and July, 2018. Three sampling sites were selected for each river with intervals of 500 m. Water samples were collected from the surface layer of the two rivers. The water samples were stored in a sealed polyethylene bucket after large particles of solids and plankton were removed with a net (mesh size of 10 μm). Sediment samples were collected using a beaker-type sampler. Water and sediment samples were stored at 4°C and transferred to the laboratory immediately for subsequent analysis. All water bodies and the riparian areas are public ground. We confirm that no permission was required for the water and sediment sampling.

### Analytical procedures for water and sediments characteristics

HQ30d Hach portable meter (Hach, USA) was used to measure dissolved oxygen (DO) on sites. Total phosphorus (TP), total nitrogen (TN), ammonium nitrogen (NH_4_^+^-N), nitrate nitrogen (NO_3_^—^N), and chemical oxygen demand (COD) were analyzed according to Methods for the Analysis of Water and Wastewater [22]. TN, TP, NH_4_^+^-N and NO_3_^—^N were measured by UV-Visible spectrophotometer (UV2450, Shimadzu, Japan). Total organic carbon (TOC) content was determined by TOC analyser (vario TOC, elementar, Germany). Sediment TP, TN, TOC, NH_4_^+^-N, and NO_3_^—^N concentrations were measured after extraction with 2 M KCl (1:5 wt/vol). All samples ran in triplicate.

### Spectroscopic characterization of sediment dissolved organic materials

Dissolved organic materials (DOM) were extracted by mixing ground and sieved freeze-dried sediment (100 mesh sieves) and Milli-Q water in a 1:10 ratio, shaking 24 h at 200 rpm, 25°C, then centrifuged at 8000 rpm for 5 min. The supernatant was filtered through 0.45 μm membrane to get DOM.

Three-dimensional excitation–emission matrix (3D-EEM) fluorescence spectra was analyzed using Horiba F-7000 (Hitachi, Japan) fluorescence spectrophotometer with an excitation (Ex) range from 200 to 450 nm and an emission (Em) range from 250 to 550 nm. The spectra were recorded at a scan rate of 12,000 nm/min, using excitation and emission slit bandwidths of 5 nm. Rayleigh scattering was subtracted from the original EEM data. Fluorescence spectra of Milli-Q water was run under identical conditions to eliminate the effect of Raman scattering. The data of fluorescence spectra were plotted by OrginPro 2017.

Six components were analyzed through 3D-EEM fluorescence spectra. C1 (Ex / Em ≤ 230 (285) / 340 nm) represents amino acid associated tryptophan-like components. C2 (Ex / Em ≤ 240 (350) / 468 nm) represents a typical terrestrial humic-like substance. C3 (Ex / Em ≤ 230 / 420 nm) represents agricultural-soil-derived humic-like or fulvic-like materials. C4 (Ex / Em ≤ 230 / 380 nm) represents a microbial humic-like substance. C5 (Ex / Em ≤ 230 (275) / 316 nm) and C6 (Ex / Em ≤ 230 (270) / ≤ 300 nm) represent those of redshifted tyrosine and typical tyrosine substances, respectively [23].

### Potential rates of denitrification and anammox

The potential rates of denitrification and anammox were measured by incubation experiments using the ^15^N isotope pairing technique and the Membrane Inlet Mass Spectrometer (MIMS) (Bay Instruments, Easton, MD) determination of ^29^N_2_ and ^30^N_2_ in the sediment slurry and overlying water [24]. Fresh sediments were mixed with helium (He)-purged water at a ratio (sediment/water) of 1:5 in 12 mL glass vials (Exetainer, Labco, U.K.) to get sediment slurries. Sediment slurry *and* overlying water were then incubated for 3 days at 20°C, 150 rpm to eliminate nitrate and oxygen. After that, the vials were spiked with 100 μL He-purged solution of ^15^NO_3_^-^ (99.2% ^15^N) to a final concentration of 100 μM ^15^N, respectively. Sediment slurry and overlying water were incubated at 20°C for 36 h and stopped by adding 200 μL saturated ZnCl_2_ solution, then the concentrations of ^29^N_2_ and ^30^N_2_ were determined by MIMS.

Both anammox and denitrification generated ^29^N_2_. Thus, the respective contributions of each process to the total ^29^N_2_ production were quantified by Eq (1):

$$P_{29}=A_{29}+D_{29}$$ (1)

where, *P*_29_, *A*_29_, and *D*_29_ represent the production rate of total ^29^N_2_, ^29^N_2_ from anammox, and ^29^N_2_ from denitrification, respectively. Because the ^28^N_2_, ^29^N_2_, and ^30^N_2_ generated from denitrification follow random isotope pairing, *D*_29_ can also be estimated by Eq (2):

$$D_{29}=P_{30}\times 2\times \left(1-F_{n}\right)\times F_{n}{}^{-1}$$ (2)

where *P*_30_ is the total ^30^N_2_ production rate, and *F*_n_ is the mole fraction of ^15^N in the nitrate pool. Consequently, the potential rates of anammox and denitrification were estimated by the following equations:

$$D_{\text{total}}=D_{29}+2\times D_{30}$$ (3)

$$A_{29}=P_{29}-D_{29}$$ (4)

where *D*_total_ and *A*_29_ are the potential rates of denitrification and anammox, respectively.

### DNA extraction and quantitative PCR assay

DNA was extracted from freeze-dried sediment (approximately 0.5 g) or 200 mL overlying water filtered through 0.22 μm filter using a FastDNA SPIN Kit for Soil (MP Biomedicals, Solon, OH, USA) according to the manufacturer’s protocol. The DNA concentration and quantity were determined using a NanoDrop 2000 spectrophotometer (Thermo Fisher Scientific, Schwerte, Germany).

Quantitative PCR (qPCR) assays were conducted using the SYBR-Green approach on ABI 7500 Real-Time PCR System (Applied Biosystems, Foster City, USA). The abundances of anammox bacteria, nitrifier (AOB), denitrifier, and total bacteria were quantified targeting the corresponding specific genes. Details about the primer sets, thermal profiles, and experimental procedures are provided in S1 Table.

### 16S rRNA gene high-throughput sequencing and data analysis

The V3-V4 hypervariable region of sample DNA were amplified using 341F/806R primer pair (341F: 5′-CCTAYGGGRBGCASCAG -3′, 806R: 5′-GGACTACNNGGGTATCTAAT-3′) and sequenced using PE250 (Illumina, CA). The sequence analysis was carried out using QIIME (version 1.9.1). The OTUs were assigned to a set of hierarchical taxa using the ribosomal database project (RDP) classifier. The α-diversity metrics, including Chao 1, OTU richness, Shannon and Simpson index were calculated to compare the bacterial richness and evenness among different treatments. Principal coordinates analysis (PCoA) of Weighted-Unifrac distance and non-metric multidimensional scaling (NMDS) analysis of Bray-Cutris distance were carried out using R (version 3.5.1) package Vegan.

The bacterial taxa (e.g., genera or species) were classified to different functional groups using the functional annotation of prokaryotic taxa (FAPROTAX) database (http://www.zoology.ubc.ca/louca/FAPROTAX/). The Phylogenetic Investigation of Communities by Reconstruction of Unobserved States (PICRUSt) was used to predict the function of bacterial community [25].

The linear discriminant analysis (LDA) effect size algorithm (LEfSe) was applied to explore the statistically significantly features of microbial communities between different samples. An alpha = 0.05 was used in Wilcoxon rank sum test, and threshold for the LDA analysis was set to be 4.0 [26].

### Statistical analysis

Statistical analysis of significant difference was analyzed by Graphpad prism 7.0 software with two-way analysis of variance (ANOVA). The level of p < 0.05 was considered statistically significant. Data were expressed as mean ± SD. Spearman correlation analysis was performed to evaluate the correlations among the potential N removal rates, relative abundances of the functional genes, and environmental factors using the SPSS 19.0 package.

## Results

### Water quality and sediment property in urban river

**Fig 1:**
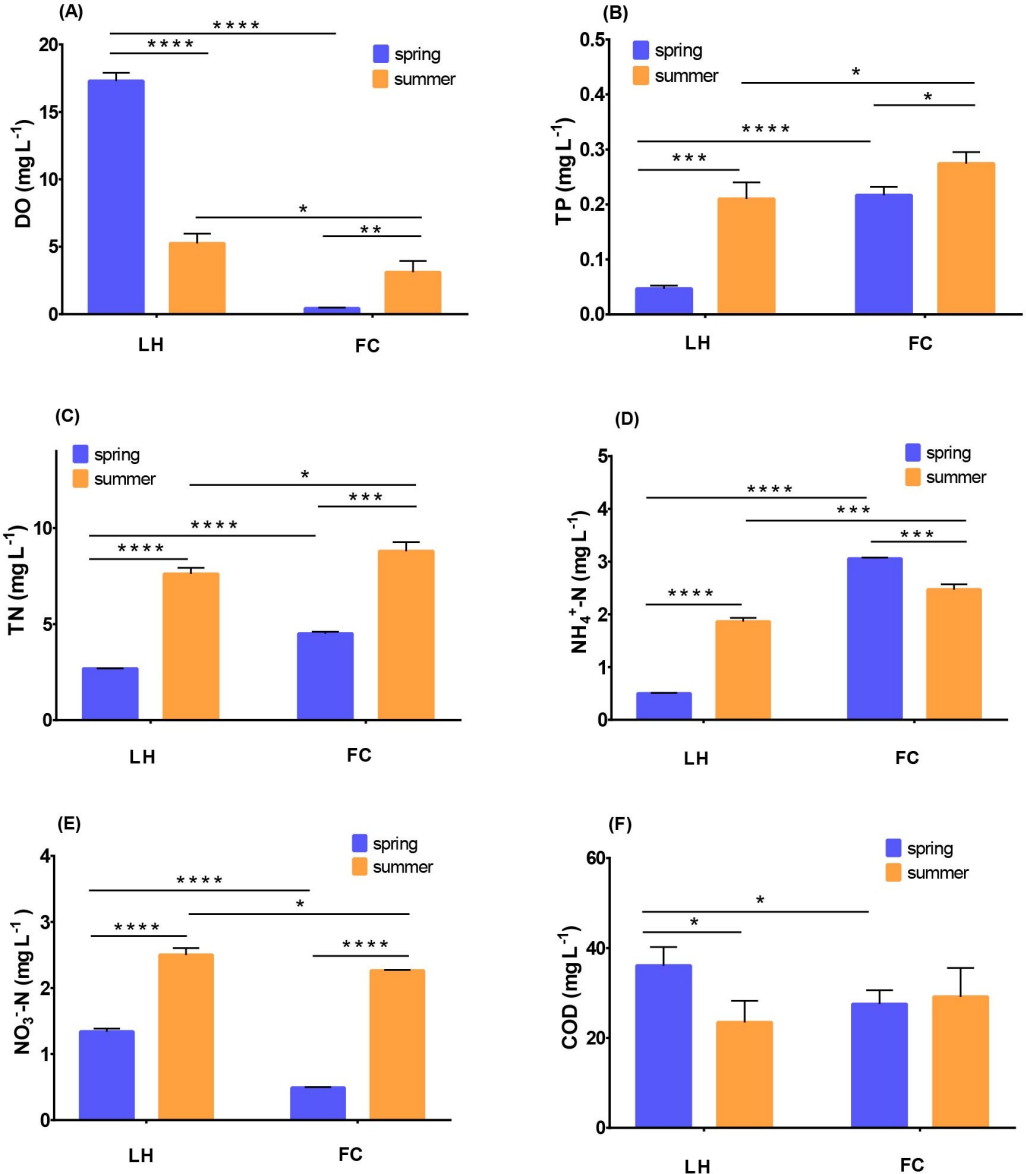
The different water quality index between FC and LH in different seasons, (A) DO, (B) TP, (C) TN, (D) NH_4_^+^-N, (E) NO_3_^—^N, (F) COD.

High concentrations of TN (4.51–8.79 mg L^-1^), TP (0.22–0.27 mg L^-1^), NH_4_^+^-N (2.47–3.06 mg L^-1^) and COD (27.55–29.15 mg L^-1^) were observed in FCW (FC overlying water). In LHW (LH overlying water), the concentrations of TN, TP, NH_4_^+^-N and COD were 2.68–7.61 mg L^-1^, 0.05–0.21 mg L^-1^, 0.50–1.86 mg L^-1^ and 23.47–36.09 mg L^-1^, respectively. In the overlying water, the concentrations of TN, TP, and NH_4_^+^-N in FCW were 1.15–1.68 times, 1.31–4.64 times and 1.33–6.11 times of their counterparts in LHW in spring and summer, respectively, whilst NO_3_^—^N acted oppositely (Fig 1). In both rivers, the concentrations of TN, TP, and NO_3_^—^N increased sharply in summer, however, NH_4_^+^-N in FCW decreased in summer (Fig 1).

**Fig 2:**
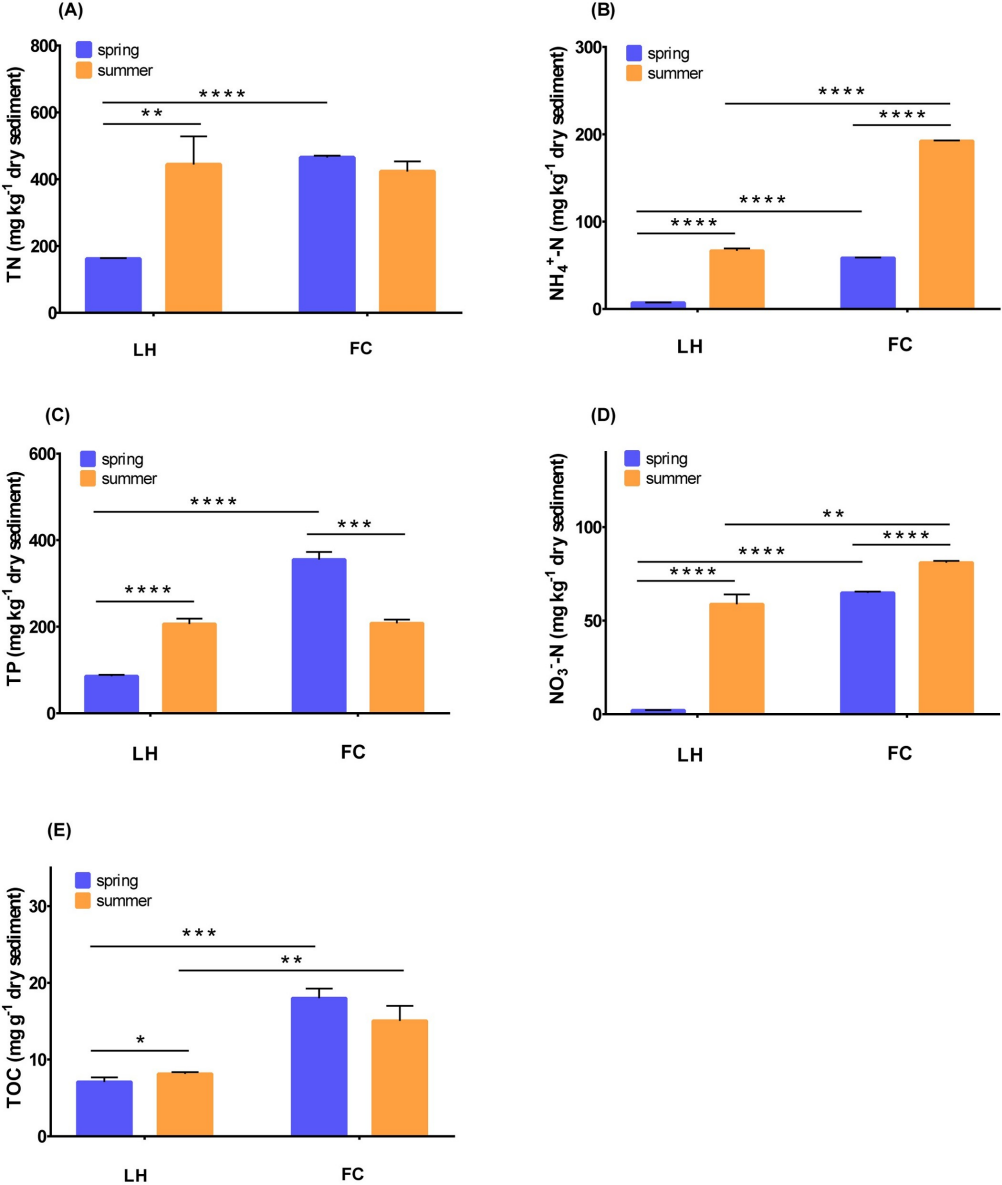
The different sediment component content between FC and LH in different seasons, (A) TN, (B) NH_4_^+^-N, (C) TP, (D) NO_3_^—^N, (E) TOC.

In sediments, the contents of TN, TP, NH_4_^+^-N, and NO_3_^—^N in FCS (FC sediment) were also significantly higher than LHS (LH sediment) (*p* < 0.05) (Fig 2), indicating that more pollutants accumulated in urban center river sediment compared with the suburban one. Despite the significant spatial variability of nutrient contents between urban and suburban rivers, both rivers showed obvious seasonal characteristics. In LHS, the contents of TN, TP and TOC in summer were significantly higher than spring (*p* < 0.05), which was consistent with the higher load as observed in overlying water in summer. In FCS, the content of TN stayed stable from spring to summer, whereas the contents of NH_4_^+^-N and NO_3_^—^N in FCS were 3.25 and 1.24 times of their spring level (Fig 2). Furthermore, TP content in FCS decreased in summer.

3D-EEM of sediment DOM showed that tryptophan-like (C1) substance in FCS (22.33% in spring, 25.93% in summer) was higher than that in LHS (15.51% in spring, 10.07% in summer). Furthermore, the data also showed that tryptophan-like (C1) substance in FCS significantly increased from spring to summer (Table 1), indicating that more partially-degraded organic matter accumulated in FCS than LHS, especially in summer. Consistent with the increase of tryptophan-like substance, the ratio of tryptophan to tyrosine increased from 5.58 to 6.57 in FCS from spring to summer (S2 Table), suggesting that more fresh protein-like organics accumulated in summer in FCS. In contrast to FCS, LHS had higher proportion of humic- or fluvic-like substance (C3), as well as microbial humic-like substance (C4) (Table 1). Both C3 and C4 components in LHS increased from spring to summer, which demonstrated that more recalcitrant DOM accumulated in LHS, especially in summer.

**Table 1:** Six components concentration (RU) through 3D-EEM fluorescence spectra in FCS and LHS.

	Substances	FC-spring	Proportion	FC-summer	Proportion	LH-spring	Proportion	LH-summer	Proportion
C1	amino acid associated tryptophan-like components	1.8925	22.33%	2.2623	25.93%	0.9929	15.51%	0.7671	10.07%
C2	terrestrial humic-like substance	4.5253	53.39%	3.8144	43.71%	3.4706	54.22%	3.3881	44.48%
C3	agricultural-soil-derived humic-like or fulvic-like materials	0.5071	5.98%	0.7899	9.05%	0.5257	8.21%	1.0726	14.08%
C4	microbial humic-like substance	0.3379	3.99%	0.6227	7.14%	0.4523	7.07%	1.0607	13.93%
C5	redshifted tyrosine	0.8732	10.30%	0.8926	10.23%	0.6306	9.85%	0.8594	11.28%
C6	tyrosine substances	0.3392	4.00%	0.3443	3.95%	0.3287	5.13%	0.4686	6.15%

### Functional genes associated with potential nitrogen removal

**Fig 3:**
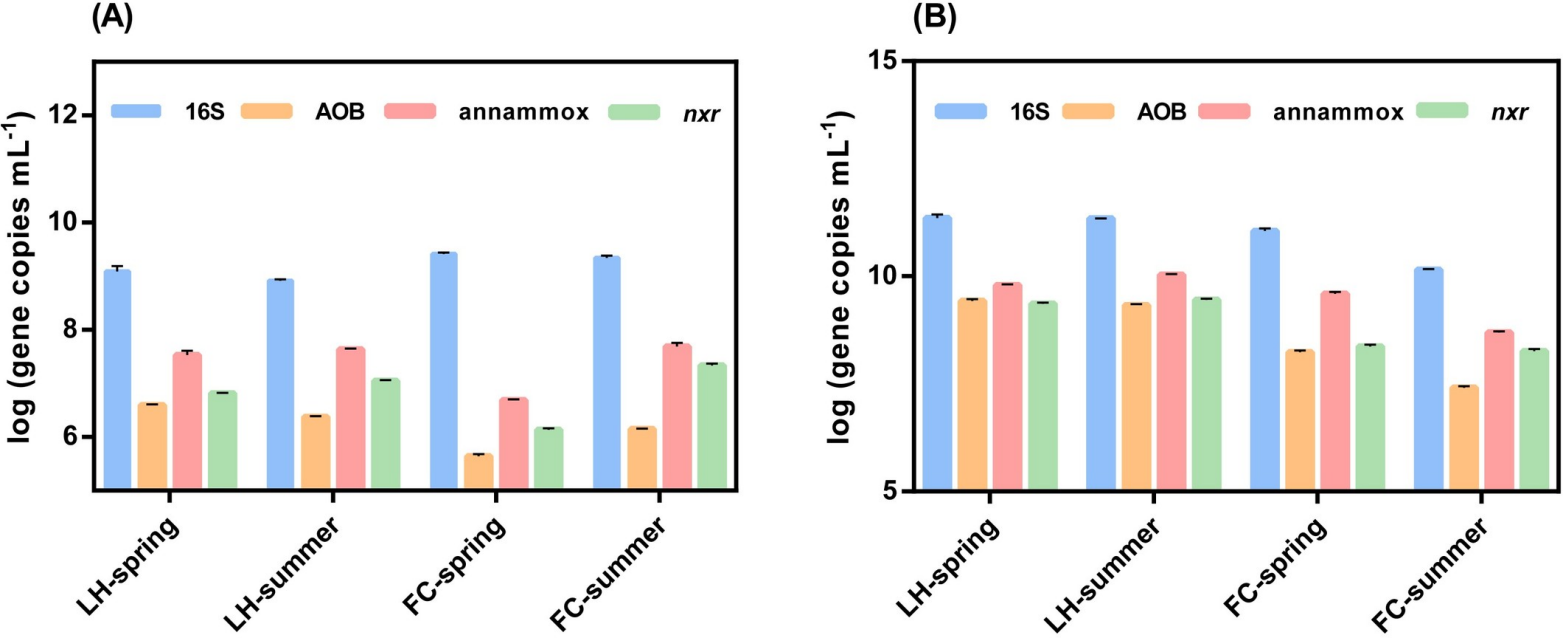
The abundance of 16S, AOB, anammox bacteria and *nxr* gene in overlying water (A) and sediment (B) in different seasons of FC and LH.

The abundances of functional genes related to potential N removal, including nitrification (*amoA*, *nxr*), denitrification (*narG*, *napA*, *nirS*, *nirK*, *norB*, and *nosZ*), and anammox 16S rRNA genes were analyzed using qPCR. In overlying water, *amoA* abundance in FCW was significantly lower than LHW (*p* < 0.05). While the abundance of *amoA* in LHW significantly decreased from spring to summer, *amoA* abundance in FCW increased from spring to summer, coinciding with the elevated DO concentration in summer FCW (Fig 1). Abundance of anammox and *nxr* genes significantly increased from spring to summer in both FCW and LHW, and the highest levels of anammox (7.62–7.75 log gene copies mL^-1^, 0.11% of total bacteria) and *nxr* (7.30–7.37 log gene copies mL^-1^, 0.05% of total bacteria) were detected in summer FCW (Fig 3). In overlying water, the abundance of all denitrification genes in spring were higher than summer in both rivers, except for *nirK* in FCW.

**Fig 4:**
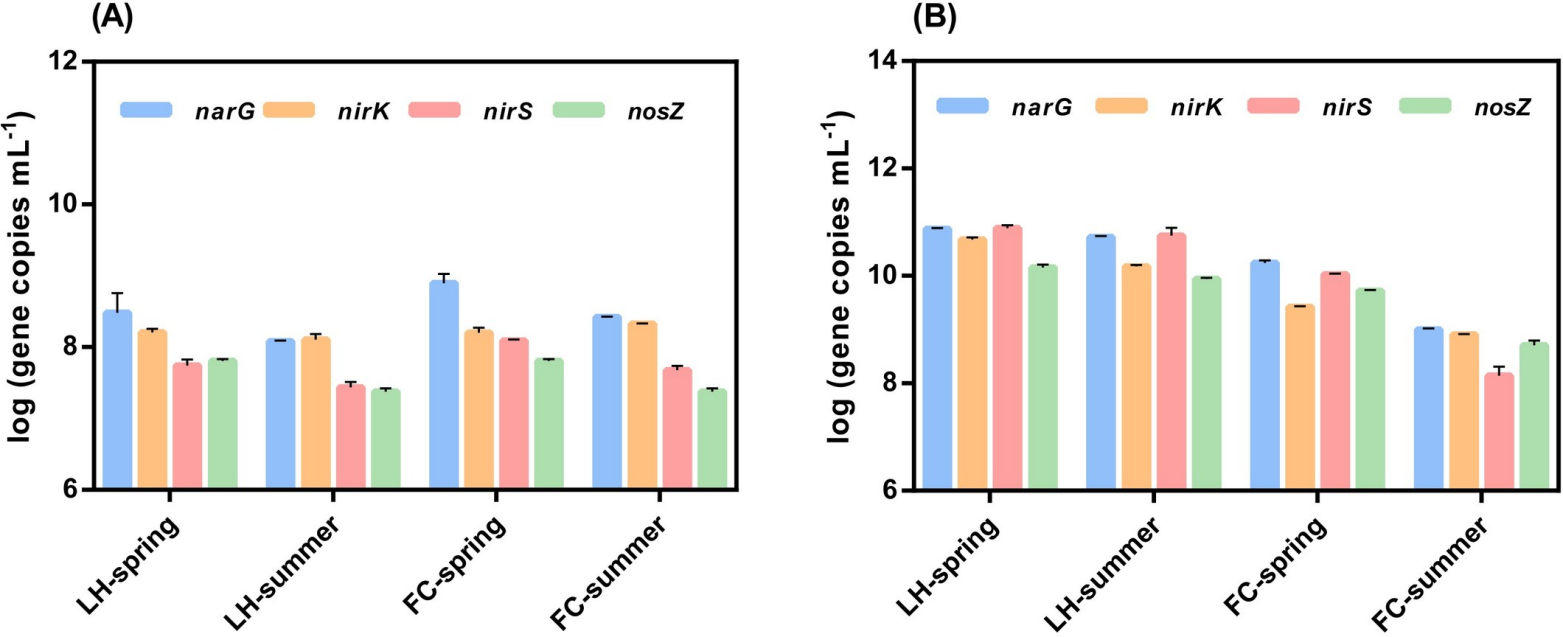
The abundance of denitrifiers genes in overlying water (A) and sediment (B) in different seasons of FC and LH.

In sediments, the abundance of *amoA*, anammox and denitrification genes was higher than overlying water (Figs 3 and 4), demonstrating that sediment was a hotspot of N metabolisms and removal. Compared with LHS, the abundance of *amoA*, anammox and denitrification genes were significantly lower in FCS (Figs 3 and 4). Just as that observed in overlying water, the abundance of denitrification genes significantly decreased in summer in both rivers (Fig 4).

### Characteristics of bacterial communities in urban river

Overall, the α-diversity characterized by Chao 1 and Shannon H indices showed that sediment bacterial communities was more diverse than their counterpart in overlying water (Table 2). This result was not surprising since the microenvironment in sediment was more heterogeneous than overlying water and niche selection played an important role in shaping bacterial diversity. In sediment, the α-diversity of LHS in summer was higher than spring, however, FCS had a rather stable α-diversity in both seasons. No matter in overlying water or sediment, the α-diversity of bacterial communities in FC were lower than in LH. In agreement with our results, a previous study showed that the food-web of urban river was low in diversity due to the greater influence of water quality degradation caused by human activities [16].

**Table 2:** The α-diversity analysis metrics of bacterial community in FC and LH.

		River	Chao 1	Shannon H	Simpson
Overlying water	Spring	LH	691	6.43	0.952
		FC	555	3.53	0.782
	Summer	LH	1477	7.28	0.983
		FC	1475	7	0.973
Sediment	Spring	LH	2798	7.47	0.976
		FC	2600	7.05	0.968
	Summer	LH	4123	9.99	0.997
		FC	2667	8.11	0.972

**Fig 5:**
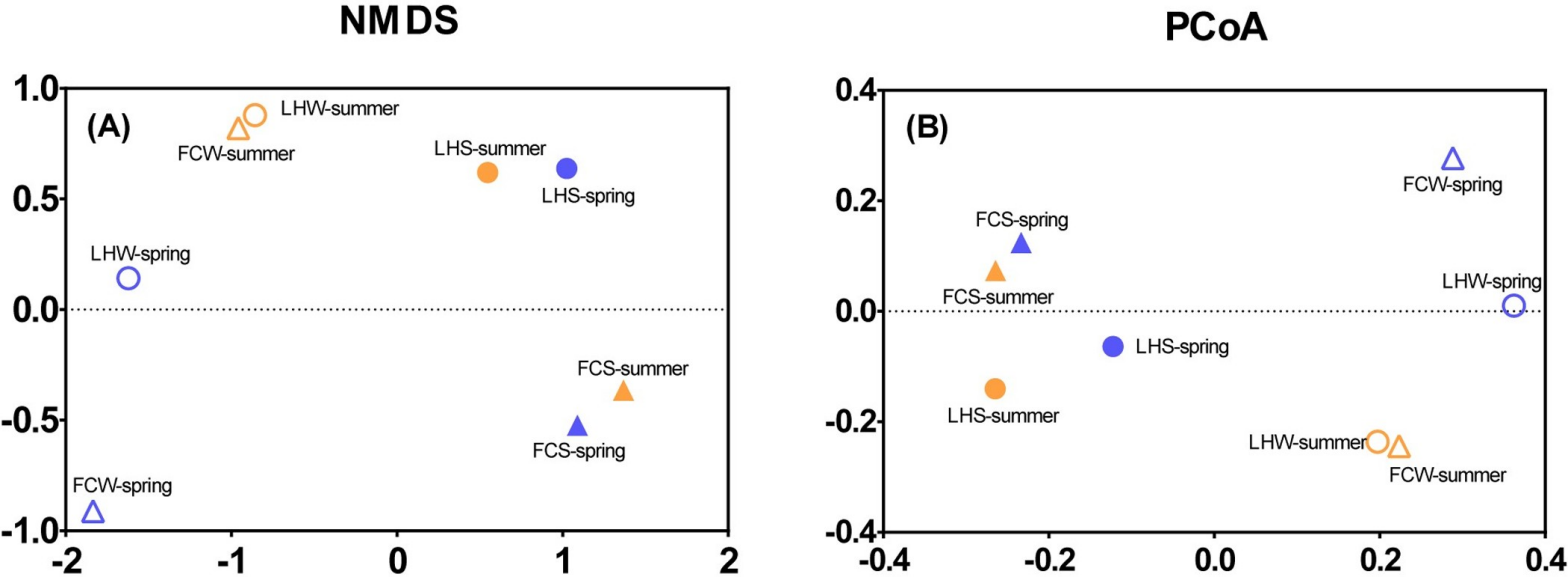
NMDS (A) and PCoA (B) analysis at the OUT level.

Beta-diversity (NMDS and PCoA) clearly revealed that bacterial communities in overlying water were separated from the benthic ones (Fig 5). Bacterial communities in overlying water clustered according to seasonal variation, however, benthic communities clustered according to sites (Fig 5). The separation of LH and FC benthic communities might be attributed to the niche differentiation between FC and LH sediment (Fig 2 and Table 1), indicating that pollutants had more legacy effect on benthic communities compared with aquatic communities.

**Fig 6:**
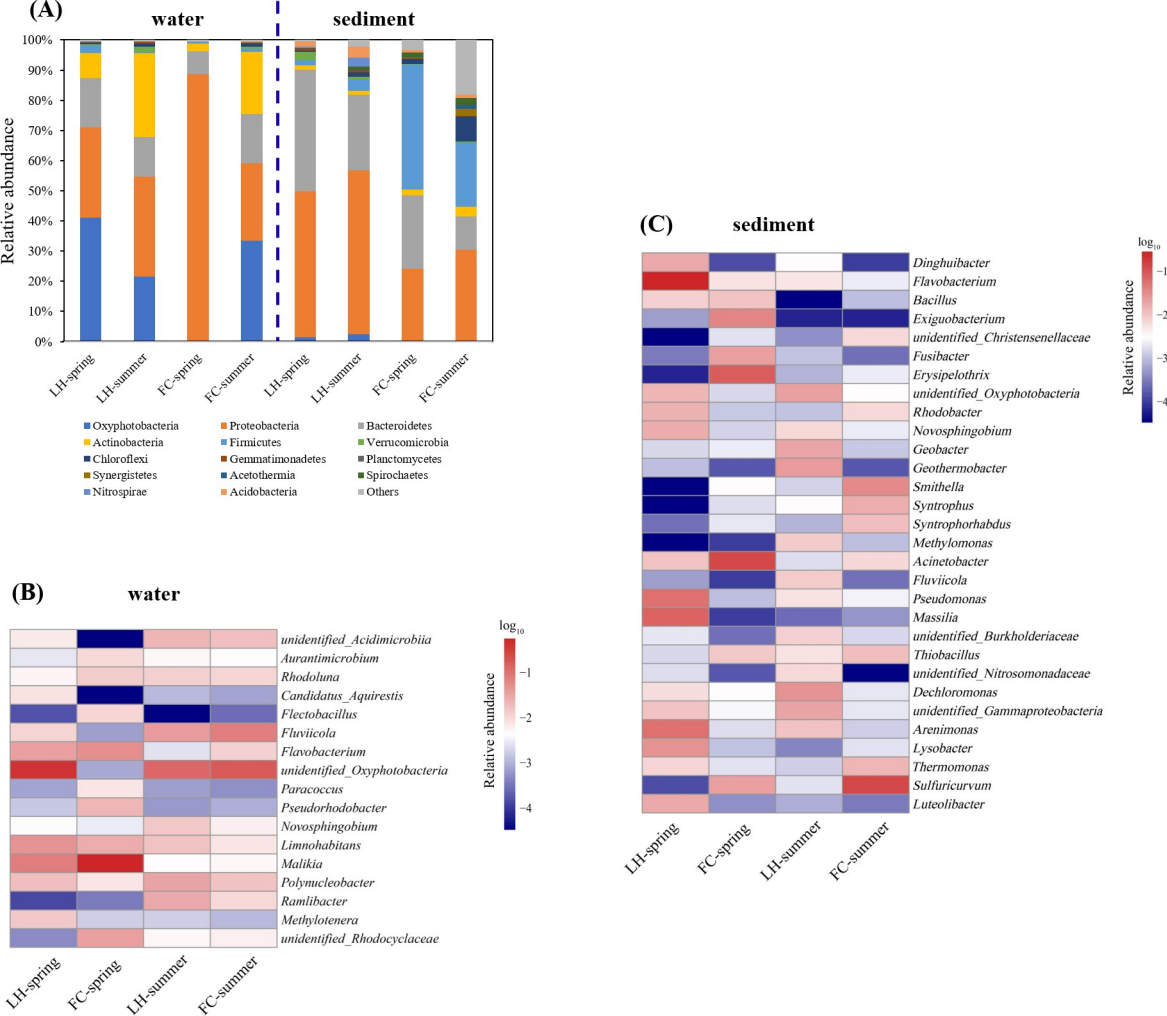
(A) Bacterial community structures at the phylum level in FC and LH; (B) Heatmap of the core bacterial communities in water at genus level. The scale bar shows the relative abundance (log_10_ scale) of each genera; (C) Heatmap of the core bacterial communities in sediment at genus level. The scale bar shows the relative abundance (log_10_ scale) of each genus.

**Fig 7:**
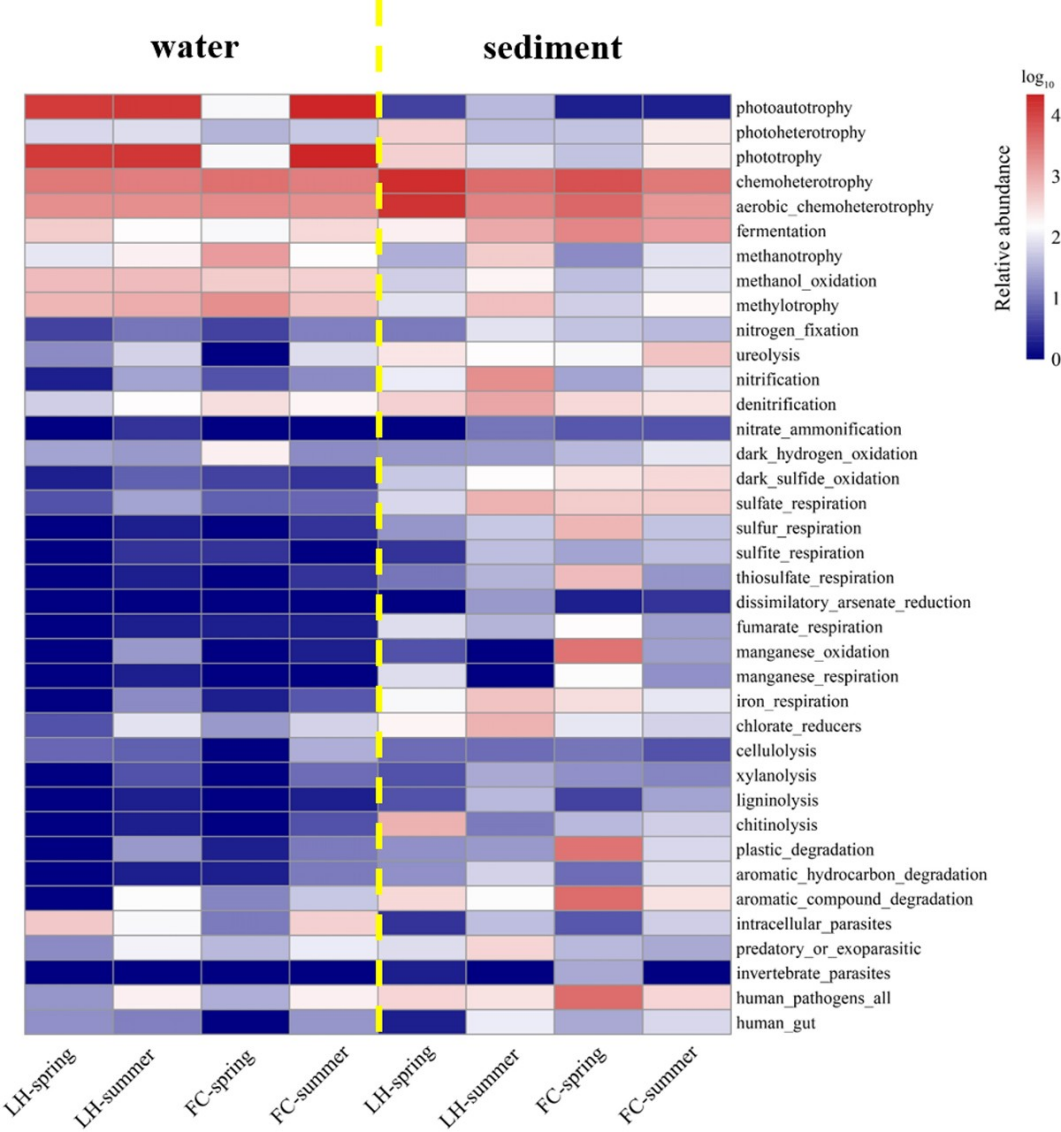
Heatmap analysis of predicted functions based on detected bacterial taxa in different sites and seasons.

A total of 36 and 58 phyla were identified in aquatic and benthic communities, respectively. For aquatic communities, 7 phyla presented relative abundance higher than 1%, and Oxyphotobacteria, Proteobacteria, Bacteroidetes and Actinobacteria jointly comprised of over 95% of all aquatic sequences in each sample (Fig 6A). Typical water born genera, including *Rhodoluna*, *Polynucleobacter*, *Fluviicola*, and *Limnohabitans* dominated in aquatic communities of both rivers (Fig 6B). These water born genera are aerobic anoxygenic phototrophs (AAPs), using acetate and other low molecular weight photoproducts, or degradation of high molecular weight compound algae exudates [27–29]. An *unidentified_Oxyphotobacteria*, a bacterial methylotroph (*Methylotenera*) and organic degraders (*Pseudorhodobacter*, *Flavobacterium*, *Fluviicola*, and *Novosphingobium*) also dominated in both rivers (Fig 6B). Although heterotrophic processes are considered to prevail in freshwater rivers and lakes with high terrestrial loading and a high content of dissolved organic carbon [30], function analysis of bacterial communities by PICRUSt showed that photosynthesis and methyl-oxidization were the dominant function in overlying water in both rivers (Fig 7). In the meantime, we found that the relative abundance of some denitrifiers, such as *Malikia*, *Flavobacterium*, and *Rhodocyclaceae*, decreased in summer, especially in FC (Fig 6B), indicating the potential of denitrification decreased in summer.

Benthic communities of LH were dominated by Proteobacteria and Bacteroidetes, which accounted for 88.1% and 77.2% of the total sequences in spring and summer, respectively (Fig 6A). Proteobacteria, Bacteroidetes, and Firmicutes dominated in FCS community (Fig 6A). The high abundance of Firmicutes (40.9% and 20.5% in spring and summer, respectively) in FCS might be attributed to the high level of organic pollution and low DO (Fig 2) as members in Firmicutes have been known for fermenting organic matters anaerobically and producing volatile organic acids [31, 32]. The predominant taxa in FCS community, such as *Novosphingobium*, *Acinetobacter*, *Flavobacterium*, *Geobacter*, and *Dechloromonas* were also identified in other heavily polluted urban river sediments and anaerobic active sludge (Fig 6C) [31, 33].

**Fig 8:**
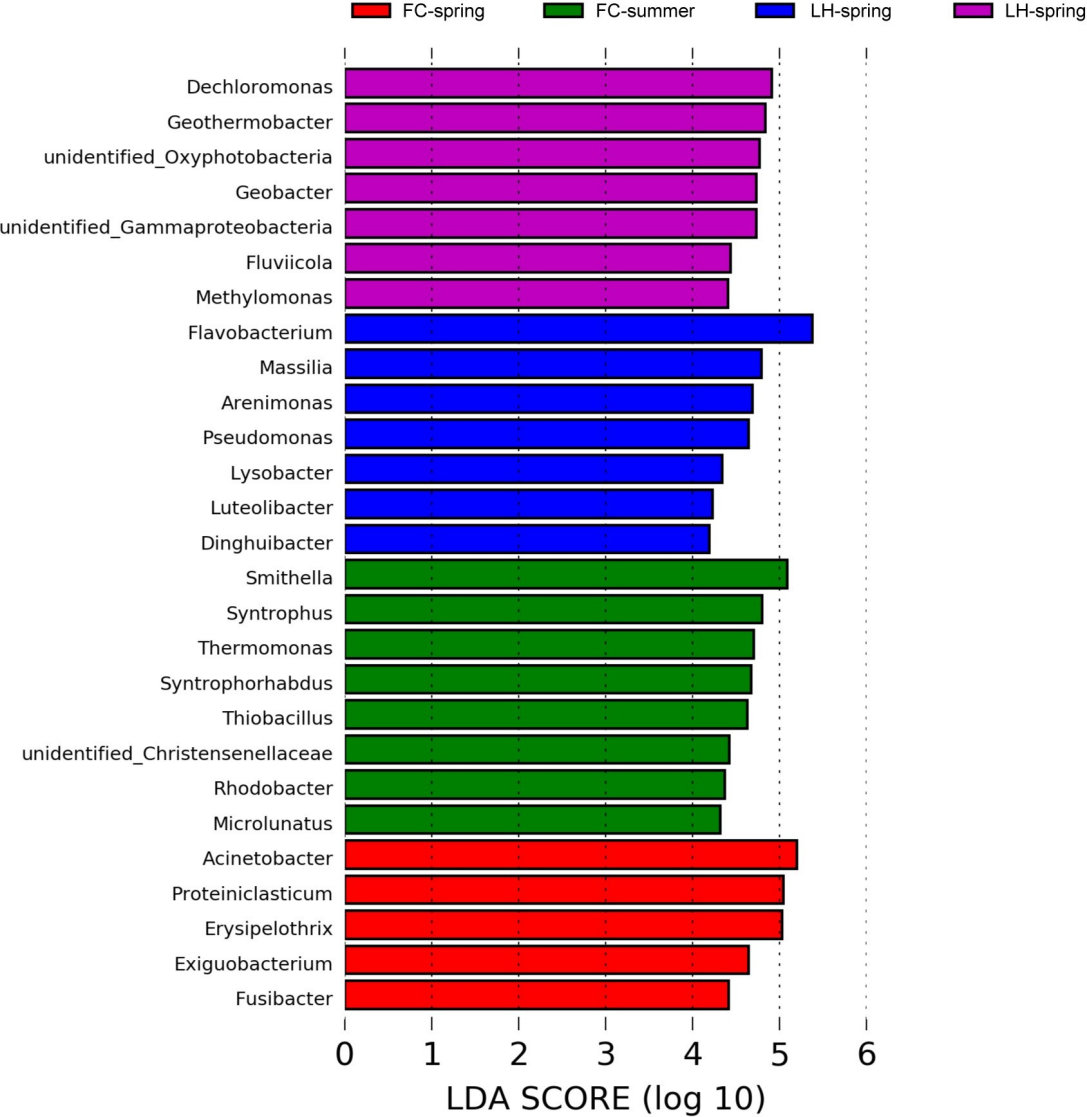
Analysis of the sediment dominant taxa based on LEfSe algorithm in FC and LH.

In benthic communities of FC and LH, LEfSe analysis identified 27 biomarkers at an LDA threshold of 4.0 (Fig 8). The summer FCS community had the greatest abundance of statistically unique taxa. Most of the marker taxa in summer FCS, such as *Smithella*, *Syntrophus*, *Thermomonas*, *Syntrophorhabdus*, *Thiobacillus*, *Christensenellaceae*, and *Rhodobacter* (Fig 6C), could degrade diverse organic matters to acetate [34, 35]. In the spring LHS, a large portion of organic degraders and heterotrophic denitrifiers were identified as the marker phylotype, including *Flavobacterium*, *Massilia*, *Arenimonas*, *Pseudomonas*, and *Lysobacter*, while the summer LHS taxa were dominated by *Dechloromonas*, *Geothermobacter*, *Oxyphotobacteria*, *Geobacter*, *Fluviicola*, and *Methylomonas* (Fig 8). As predicted by PICRUSt, the sediment core communities had higher levels of predicted functions involved in fermentation, anaerobic respiration (such as sulfate, arsenate, fumarate, manganese, and iron), and complex-carbon compound degradation (Fig 7). More fermentation, sulfate reduction and metal oxidization and reduction function were found in FCS community (Fig 7).

### Potential nitrogen removal rate in urban river and its determining factors

**Fig 9:**
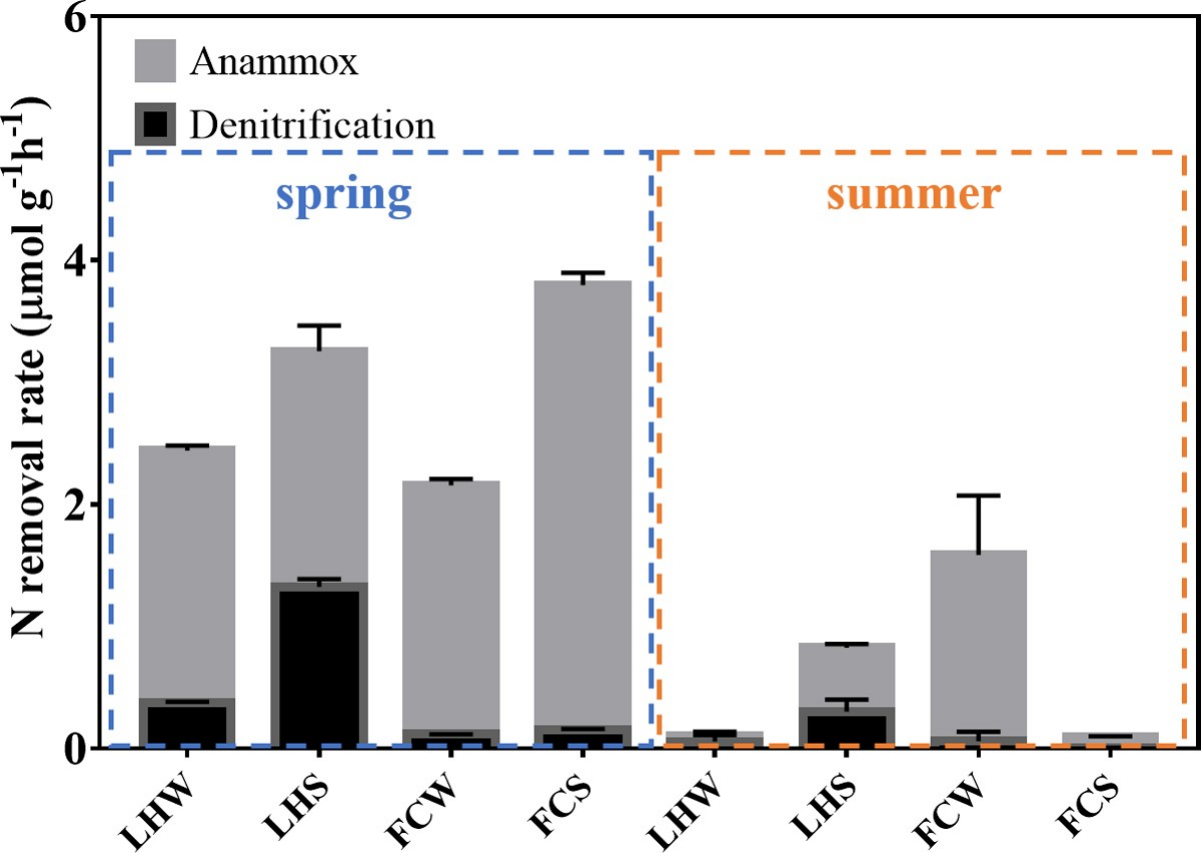
Potential rates of denitrification and anammox in overlying water and sediment of FC and LH in different seasons.

Laboratory ^15^N tracer techniques are often used as standard methods to determine the potential rates of denitrification and anammox. This technique has been successfully applied to quantify the in situ N removal rates [24]. Our study found that the rates of anammox were significantly higher than denitrification in both rivers (*p* < 0.05) (Fig 9). The highest anammox rate was detected in the spring FCS (3.64 ± 0.10 μmol N g^-1^ h^-1^), which accounted for 95.87% of the total N removal rate. In LHS, denitrification rates (1.32 ± 0.065 μmol N g^-1^ h^-1^ in spring, 0.30 ± 0.099 μmol N g^-1^ h^-1^ in summer) were about 3–5 times higher than LHW (0.38 ± 0.006 μmol N g^-1^ h^-1^ in spring, 0.05 ± 0.004 μmol N g^-1^ h^-1^ in summer). However, the denitrification rates in FCS (0.16 ± 0.005 μmol N g^-1^ h^-1^) and FCW (0.12 ± 0.002 μmol N g^-1^ h^-1^) were similar in spring and significantly lower denitrification rates were observed in summer FCS (0.005 ± 0.002 μmol N g^-1^ h^-1^), indicating the denitrification in the sediment of FC river was severely inhibited in summer (Fig 9). These data also showed that the rates of denitrification and anammox in both rivers was significantly inhibited in summer regardless of overlying water and sediment (*p* < 0.05) compared with their spring counterpart (Fig 9). The potential denitrification rate of LHS was higher than that of FCS in spring and summer, and the potential rate of anammox in LHS was higher than FCS in summer (Fig 9).

We conducted correlation analysis on the factors affecting potential nitrogen removal rate, and the results showed that the denitrification rate in both of overlying water and sediments had a significant positive correlation with the abundance of denitrification genes (S3 and S4 Tables). NH_4_^+^-N had a significant negative correlation with both of denitrification and anammox rates in sediment (S4 Table). The diversity of bacterial communities was negatively correlated with anammox rate (*p* < 0.01) (S3 and S4 Tables). In overlying water, the potential denitrification rate was significantly positively correlated with TOC (*p* < 0.01) (S3 Table), while the potential denitrification rate was significantly negatively correlated with TOC in sediment (*p* < 0.01) (S4 Table).

## Discussion

### Characteristics of water and sediment qualities in an urban center and suburban river

Water and sediment qualities are integrated representations of multiple physical, chemical, and biological processes. Similar to other polluted urban rivers [2, 6, 16], FC river, which ran through the urban center, was characterized by high contents of TN, TP, NH_4_^+^-N, and low levels of DO (Figs 1 and 2). In the overlying water of both rivers, the concentrations of TN, TP, and NO_3_^—^N in summer were higher than spring, which might be attributed to a larger amount of surface runoff containing higher pollutants into rivers caused by rainfall in summer (674.70 mm during May to September, accounting for 64.38% of the annual rainfall in Wuxi) [36]. However, the seasonal variation of water quality in FCW was not as high as LHW (Fig 1), indicating that pollution flowing into a river in urban area was more intense and stable than in a suburb regardless of seasonal variation.

Previous studies have demonstrated that as a city develops along a river, the discharge of rough domestic wastewater results in high concentrations of degradable DOM in urban rivers [37, 38]. Our results of 3D-EEM analysis of sediment DOM indicated that more “fresh” protein-like organics accumulated in FCS, whilst more recalcitrant DOM existed in LHS (Table 1). Thus, the high proportion of protein-like organics might explain the high TN and TP contents in FCW, meanwhile, degradation of protein-like organics would simultaneously release NH_4_^+^-N by ammonification process and exhaust DO in FCW (Fig 1). Our data also showed that the concentration of NO_3_^—^N in FCW was lower than LHW (Fig 1), which might be partly attributed to the low DO in FCW (Fig 1) for O_2_ is the essential substrate of the nitrification process. The slow transformation of NH_4_^+^-N to NO_3_^—^N subsequently resulted in N accumulation in FC for nitrification was normally considered as the first step of N removal [13].

In sediments, although almost all of the nutrient levels of both rivers increased in summer, we found that TP significantly decreased in FCS (Fig 2). Linear regression analysis showed that contents of sediment TN and TP had a significantly positive relationship with TN and TP levels in overlying water (S1 Fig), indicating the mutual influence of water and sediment qualities. The combination of inflow pollutants and microbial driven biogeochemical processes might explain the difference between FC and LH rivers. Sediments not only act as sink and source of carbon and nutrients, but are also is the hotspots of biogeochemical processes. Firstly, the inflow pollutants into river caused different substance suitable for microbes and changed the environment in sediments. In line with a previous report, protein-like DOM enriched in FC, the river located in an urban center [38], whereas microbially-soil-derived humic-like DOM enriched in LH, the suburban river surrounded by wetlands [38]. The protein-like DOM was mainly mineralized to CO_2_, which would have consumed the DO in sediments [38]. Thus, the high proportion of biodegradable fresh protein-like organics in FC river sediment might have stimulated microbial activities and caused oxygen depletion in sediment [39], furtherly influencing the biogeochemical processes that control nutrient cycling [40]. Subsequently, internal feedbacks were initiated, including phosphorus release from the sediment [19], reduction in activities of nitrification and denitrification [40, 41].

### Difference of potential nitrogen removal pathway and functional gene abundance in an urban center and suburban river

In this study, we used ^15^NO_3_^-^ to quantify the potential rates of denitrification and anammox in both of overlying water and sediments. The results showed that the potential rates of denitrification were significantly lower than anammox in both rivers and both seasons (Fig 9). The low level of denitrification and high proportion of anammox processes indicated that anammox might be the main potential N removal process in the two rivers we studied, especially in the urban center river. Previous studies have also evidenced the widespread distribution of anammox and the significant contribution of anammox to N removal in many aquatic ecosystems [42–44]. In marine sediments, anammox could account for 24–67% of N loss [45, 46] and in the Black Sea and Gulfo Dulce, 20–40% of N loss in the suboxic water columns was attributed to anammox [46–48]. In both rivers, the rates of denitrification were lower than that in constructed wetlands [44], whereas the denitrification rates were comparable or even higher than that in riparian sediment (about 0.011 μmol ^15^N g^-1^ dry soil h^-1^) [49]. Dalsgaard et al. have demonstrated that anammox tolerates higher oxygen concentrations than denitrification [50]. We speculated that under the same oxygen environment, anammox has a higher rate than denitrification. Meanwhile, high concentration of NH_4_^+^-N in the two rivers is conducive to the occurrence of anammox.

The rates of anammox and denitrification were severely inhibited in summer in both rivers, which might explain the sharp increase of TN in summer besides the heavier inflow pollutant (Figs 1 and 2). As discussed above, the hypoxia in summer resulted in the reduction of potential N removal activities by inhibiting nitrification processes [41]. Our results showed that the abundance of all functional genes related to potential N removal significantly decreased in summer (Fig 4). These combined results indicated that the propagation of the bacteria harboring these functional genes and activities of nitrification, denitrification and anammox might be inhibited in summer, which subsequently slow down the potential N removal in summer, and exacerbate the accumulation of N in rivers.

### Difference of bacterial communities in an urban center and suburban river

Our data showed that there was no significant difference in the diversity and bacterial community compositions in the overlying water of FC and LH rivers although the studied characteristics of water quality were significantly different between rivers and seasons. Zhao et al. [16] analyzed 39 stations around Jinan city with different water quality and identified hydraulic parameters, temperature, transparency, electrical conductivity, and dissolved oxygen, instead of water quality, as the main driving factors of aquatic communities. Sánchez-Carrillo [51] studied 10 lakes around the world and found temperature and altitude were the important driving factors of food-web structure. Our results and these previous studies combined indicated that the diversity, composition and functions of aquatic bacterial communities were more driven by physical environmental factors instead of water quality, which explained the similarity of aquatic bacterial communities between FCW and LHW (Fig 5). The results of beta-diversity analysis about the way of the bacteria communities gathered was interesting. We consider that the overlying water was fluid and directly related to human seasonal variation of using water. Therefore, bacterial community in overlying water was aggregated according to seasonal variation. The sediments in the river changed little compared with the overlying water, and the sediments were relatively stable. Therefore, the stable benthic microbial community was formed, showing that the benthic bacterial community gathered according to the location.

Differently structured bacterial communities differed in their metabolic substance, degradation pattern and production, and community composition often directly influenced ecosystem function [52]. In contrast to aquatic microbial communities, sediment microbial communities differed between the FC and LH river (Fig 5). Compared with LHS, fermentation and sulfate reduction bacteria existed in high abundance in FCS (Fig 7), which might be explained by the niche selection and adaptation of such functional bacteria to the environment of high carbon and low DO content (Figs 1 and 2). The fermentative process is not effective for mineralization of organics, and the production of fermentation, such as short-chain fatty acids would contribute to the stench odor of urban river [31]. The production of FeS, H_2_S, organic sulfides, NH_3_, amines, and short chain fatty acids through sulfate reduction and fermentation was considered as the main cause of the black and stench of urban rivers [8]. In addition, the reduced sulfur might inhibit nitrification [53] and subsequent N removal process [54]. In addition, it was interesting to find that autotrophic denitrifiers, *Thermomonas* and *Thiobacillus*, instead of heterotrophic denitrifiers, dominated in FCS (Fig 6C). The autotrophic denitrifiers, *Thermomonas* and *Thiobacillus*, could reduce nitrate by coupling Fe(II) and reduced sulfur oxidation, respectively [55, 56]. However, denitrification efficiency of autotrophic denitrifiers was lower than heterotrophic denitrifiers due to their slow growth and the inadequate supply of electrons to reduce NO_x_^—^N. Therefore, we proposed that the biochemical processes in FCS might be less effective in removal of organics and N compared with LHS, resulting in the accumulation of organics and N in the urban river (Fig 2), which would then aggravate the accumulation of pollution in the river.

### Factors influencing potential nitrogen removal activities

We further analyzed the factors influencing potential N removal activities. The denitrification rate in both of overlying water and sediments showed a significant positive correlation with the abundance of denitrification genes (S3 and S4 Tables), so the low level of denitrification genes in summer could explain the corresponding sharply decreased denitrification rate. DO was not correlated with most of the functional genes and potential N removal rates, probably because N was primarily removed through anammox (S3 Table). Although NH_4_^+^-N is one of the substrates of anammox, NH_4_^+^-N had a significant negative correlation with both denitrification and anammox rates in sediment (S4 Table). The high protein-like substance content of FC led to high ammonium formation from organic matter mineralization, resulting in NH_4_^+^-N concentration of (192.4 ± 0.66) mg kg^-1^ dry sediment in FC. A previous study had reported that free un-ionized ammonia inhibited anammox at concentrations exceeding 2 mg N L^-1^ [57]. High NH_4_^+^-N concentration could stimulate the growth and activity of nitrifiers [58], however, nitrifying bacteria have been found to be more sensitive than heterotrophic bacteria to free un-ionized ammonia [59]. High concentrations of NH_4_^+^-N such as those found in FCS, have resulted in the inhibition of nitrification and anammox activity due to presence of free un-ionized ammonia [57, 60, 61]. Organic carbon and nitrate can serve as electron donors and substrates for denitrifying bacteria. The samples were amended by N when potential denitrification rate was determined in our study, the availability of C sources affected denitrification. Our results showed that the potential denitrification rate in water was significantly positively correlated with TOC, which is consistent with previous studies [24]. However, the potential denitrification rate in sediments was significantly negatively correlated with TOC, which may be related to differences in bacterial communities in sediments as above discussion.

Sometimes, higher species diversity of bacterial communities results in greater rates of many functions in ecosystems [62], but we found that diversity was negatively correlated with anammox rate (*p* < 0.01) (S3 and S4 Tables). This finding was probably due to the competition for NH_4_^+^-N with other bacteria affected the growth and gene expression of these functional bacteria related with nitrogen, which dampened the function of potential N removal. Previous studies with synthesized microcosms had demonstrated that high richness increases the inhibitory effects of competitors and dampens ecosystem functions [63, 64]. Both the overlying water and sediment of the rivers had extremely high diversity of bacterial communities in summer, where competitive or antagonistic stress associated with substrate and inter-specific interactions may contribute to the suppression of potential N removal rates in summer. Reduced removal rate of N in the urban river would have intensified N accumulation and would contribute to the continued decline in water quality.

## Conclusion

The characteristics of N transformation and underlying bacterial mechanisms was comparatively investigated in a typical urban river and a suburban river, in Wuxi, China. Our results indicated that the concentration of total nitrogen, ammonia and nitrate accumulated faster in the urban river. Consistently, the abundance of functional genes associated with N transformation in the urban river was lower. Bacterial community analysis showed that acidogenic, fermentative and sulfate reductive bacteria dominated in benthic community of the urban river. Isotopic pairing experiments further revealed that anammox was the main potential N removal pathway in both rivers and potential N removal was inhibited in the urban river. Both gene abundance and isotopic pairing experiments showed that potential N removal was inhibited in summer in both rivers. Taken together, the altered N biotransformation and bacterial communities in an urban river could further promote pollution in urban rivers.

## Supporting information

S1 Fig Linear regression analysis of the sediment TN (A) and TP (B) with the water.
(DOCX)

S1 Table Primers and programs of the target genes in the quantitative PCR analysis. (DOCX)

S2 Table The DOM component tryptophan to tyrosine ratio in FCS and LHS. (DOCX)

S3 Table Spearman's correlation coefficient of potential N removal rate with relative abundances of functional genes and environmental factors in overlying water. (DOCX)

S4 Table Spearman’s correlation coefficient of the potential N removal rate with relative abundances of functional genes and environmental factors in sediment. (DOCX)

## References

[1] Hobbie SE, Finlay JC, Janke BD, Nidzgorski DA, Millet DB, Baker LA. Contrasting nitrogen and phosphorus budgets in urban watersheds and implications for managing urban water pollution. Proceedings of the National Academy of Sciences. 2017; 114(16):4177–4182. 10.1073/pnas.1618536114 28373560 PMC5402417

[2] Song C, Liu X, Song Y, Liu R, Gao H, Han L, et al Key blackening and stinking pollutants in Dongsha River of Beijing: Spatial distribution and source identification. Journal of Environmental Management. 2017; 200:335–346. 10.1016/j.jenvman.2017.05.088 28595127

[3] Sheng Y, Qu Y, Ding C, Sun Q, Mortimer RJG. A combined application of different engineering and biological techniques to remediate a heavily polluted river. Ecological Engineering. 2013; 57:1–7. 10.1016/j.ecoleng.2013.04.004

[4] He DF, Chen RR, Zhu EH, Chen N, Yang B, Shi HH, et al Toxicity bioassays for water from black-odor rivers in Wenzhou, China. Environmental Science and Pollution Reswarch. 2015; 22(3):1731–1741. 10.1007/s11356-013-2484-1 24385189

[5] Janke BD, Finlay JC, Hobbie SE. Trees and Streets as Drivers of Urban Stormwater Nutrient Pollution. Environmental Science & Technology. 2017; 51(17):9569–9579. 10.1021/acs.est.7b02225 28756675

[6] Cai W, Li Y, Shen Y, Wang C, Wang P, Wang L, et al Vertical distribution and assemblages of microbial communities and their potential effects on sulfur metabolism in a black-odor urban river. Journal of Environmental Management. 2019; 235:368–376. 10.1016/j.jenvman.2019.01.078 30708274

[7] Pan M, Zhao J, Zhen SC, Heng S, Wu J. Effects of the combination of aeration and biofilm technology on transformation of nitrogen in black-odor river. Water Science and Technology. 2016; 74(3):655–662. 10.2166/wst.2016.212 27508370

[8] Liang Z, Siegert M, Fang W, Sun Y, Jiang F, Lu H, et al Blackening and odorization of urban rivers: a bio-geochemical process. FEMS Microbiology Ecology. 2018; 94(3). 10.1093/femsec/fix180 29293959

[9] Hamzeh M, Ouddane B, Daye M, Halwani J. Trace Metal Mobilization from Surficial Sediments of the Seine River Estuary. Water, Air, & Soil Pollution. 2014; 225(3). 10.1007/s11270-014-1878-0

[10] Weisener C, Lee J, Chaganti SR, Reid T, Falk N, Drouillard K. Investigating sources and sinks of N2O expression from freshwater microbial communities in urban watershed sediments. Chemosphere. 2017; 188(Supplement C):697–705. 10.1016/j.chemosphere.2017.09.036 28934707

[11] Jobin L, Namour P. Bioremediation in Water Environment: Controlled Electro-Stimulation of Organic Matter Self-Purification in Aquatic Environments. Advances in Microbiology. 2017; 7(12):813–852. 10.4236/aim.2017.712064

[12] Steele MK, Heffernan JB. Morphological characteristics of urban water bodies: mechanisms of change and implications for ecosystem function. Ecological Applications. 2014; 24(5):1070–1084. 10.1890/13-0983.1 25154097

[13] Kuypers MMM, Marchant HK, Kartal B. The microbial nitrogen-cycling network. Nature Reviews Microbiology. 2018; 16(5):263–276. 10.1038/nrmicro.2018.9 29398704

[14] Ibekwe AM, Ma J, Murinda SE. Bacterial community composition and structure in an Urban River impacted by different pollutant sources. Science of The Total Environment. 2016; 566:1176–1185. 10.1016/j.scitotenv.2016.05.168 27267715

[15] Archana A, Thibodeau B, Geeraert N, Xu MN, Kao S-J, Baker DM. Nitrogen sources and cycling revealed by dual isotopes of nitrate in a complex urbanized environment. Water Research. 2018; 142:459–470. 10.1016/j.watres.2018.06.004 29913387

[16] Zhao CS, Yang Y, Yang ST, Xiang H, Wang F, Chen X, et al Impact of spatial variations in water quality and hydrological factors on the food-web structure in urban aquatic environments. Water Research. 2019; 153:121–133. 10.1016/j.watres.2019.01.015 30708191

[17] Staley C, Unno T, Gould TJ, Jarvis B, Phillips J, Cotner JB, et al Application of Illumina next-generation sequencing to characterize the bacterial community of the Upper Mississippi River. Journal of Applied Microbiology. 2013; 115(5):1147–1158. 10.1111/jam.12323 23924231

[18] Lin S, Wang Y, Lin J, Quan C. Response of Planktonic and Benthic Microbial Community to Urban Pollution from Sewage Discharge in Jilin Reach of the Second Songhua River, China. CLEAN—Soil Air Water. 2015; 42(10):1376–1383. 10.1002/clen.201200328

[19] Conley DJ, Humborg C, Rahm L, Savchuk OP, Wulff F. Hypoxia in the Baltic Sea and Basin-Scale Changes in Phosphorus Biogeochemistry. Environmental Science & Technology. 2002; 36(24):5315–5320. 10.1021/es025763w 12521155

[20] Bao LL, Wang XY, Chen YJ. Abundance and distribution of ammonia-oxidizing microorganisms in the sediments of Beiyun River, China. Annals of Microbiology. 2016; 66(3):1075–1086. 10.1007/s13213-016-1191-9 23624482

[21] Bettez ND, Groffman PM. Nitrogen Deposition in and near an Urban Ecosystem. Environmental Science & Technology. 2013; 47(11):6047–6051. 10.1021/es400664b 23631416

[22] Bureau NE. Methods for the Analysis of Water and Wastewater, fourth ed. Chinese Environmental Science Press, Beijing 2002.

[23] Zhou Y, Xiao Q, Yao X, Zhang Y, Zhang M, Shi K, et al Accumulation of Terrestrial Dissolved Organic Matter Potentially Enhances Dissolved Methane Levels in Eutrophic Lake Taihu, China. Environmental Science & Technology. 2018; 52(18):10297–10306. 10.1021/acs.est.8b02163 30141916

[24] Shan J, Zhao X, Sheng R, Xia Y, ti C, Quan X, et al Dissimilatory Nitrate Reduction Processes in Typical Chinese Paddy Soils: Rates, Relative Contributions, and Influencing Factors. Environmental Science & Technology. 2016; 50(18):9972–9980. 10.1021/acs.est.6b01765 27499451

[25] Crampon M, Bodilis J, Portet-Koltalo F. Linking initial soil bacterial diversity and polycyclic aromatic hydrocarbons (PAHs) degradation potential. Journal of Hazardous Materials. 2018; 359:500–509. 10.1016/j.jhazmat.2018.07.088 30086520

[26] Yan W, Guo Y, Xiao Y, Wang S, Ding R, Jiang J, et al The changes of bacterial communities and antibiotic resistance genes in microbial fuel cells during long-term oxytetracycline processing. Water Research. 2018; 142:105–114. 10.1016/j.watres.2018.05.047 29864646

[27] Hahn MW, Scheuerl T, Jezberová J, Koll U, Jezbera J, Šimek K, et al The Passive Yet Successful Way of Planktonic Life: Genomic and Experimental Analysis of the Ecology of a Free-Living Polynucleobacter Population. PLoS One. 2012; 7(3):e32772 10.1371/journal.pone.0032772 22448227 PMC3308952

[28] Martinezgarcia M, Swan BK, Poulton NJ, Gomez ML, Masland D, Sieracki ME, et al High-throughput single-cell sequencing identifies photoheterotrophs and chemoautotrophs in freshwater bacterioplankton. The ISME Journal. 2012; 6(1):113–123. 10.1038/ismej.2011.84 21716306 PMC3246240

[29] Zheng Q, Lu JY, Wang Y, Jiao NZ. Genomic reconstructions and potential metabolic strategies of generalist and specialist heterotrophic bacteria associated with an estuary Synechococcus culture. Fems Microbiology Ecology. 2019; 95(3):13 10.1093/femsec/fiz017 30689834

[30] Ricão Canelhas M, Andersson M, Eiler A, Lindström ES, Bertilsson S. Influence of pulsed and continuous substrate inputs on freshwater bacterial community composition and functioning in bioreactors. Environmental Microbiology. 2017; 19(12):5078–5087. 10.1111/1462-2920.13979 29124844

[31] Wang X, Li Y, Zhang Y, Pan Y-R, Li L, Liu J, et al Stepwise pH control to promote synergy of chemical and biological processes for augmenting short-chain fatty acid production from anaerobic sludge fermentation. Water Research. 2019; 155:193–203. 10.1016/j.watres.2019.02.032 30849733

[32] Dai X, Yan H, Li N, He J, Ding Y, Dai L, et al Metabolic adaptation of microbial communities to ammonium stress in a high solid anaerobic digester with dewatered sludge. Scientific Reports. 2016; 6:28193 10.1038/srep28193 27312792 PMC4911566

[33] Liu X, Xu Q, Wang D, Wu Y, Yang Q, Liu Y, et al Unveiling the mechanisms of how cationic polyacrylamide affects short-chain fatty acids accumulation during long-term anaerobic fermentation of waste activated sludge. Water Research. 2019; 155:142–151. 10.1016/j.watres.2019.02.036 30844675

[34] Shi J, Zhang B, Qiu R, Lai C, Jiang Y, He C, et al Microbial Chromate Reduction Coupled to Anaerobic Oxidation of Elemental Sulfur or Zerovalent Iron. Environmental Science & Technology. 2019; 53(6):3198–3207. 10.1021/acs.est.8b05053 30776217

[35] Qiu YL, Hanada S, Ohashi A, Harada H, Kamagata Y, Sekiguchi Y. Syntrophorhabdus aromaticivorans gen. nov., sp. nov., the first cultured anaerobe capable of degrading phenol to acetate in obligate syntrophic associations with a hydrogenotrophic methanogen. Applied and environmental microbiology. 2008; 74(7):2051–2058. 10.1128/AEM.02378-07 18281436 PMC2292594

[36] Feng L, Sun X, Zhu X. Impact of floodgates operation on water environment using one-dimensional modelling system in river network of Wuxi city, China. Ecological Engineering. 2016; 91:173–182. 10.1016/j.ecoleng.2016.02.042

[37] Xu B, Li J, Huang Q, Gong Q, Li L. Impacts of land use patterns and typhoon-induced heavy rainfall event on dissolved organic matter properties in the South Tiaoxi River, China. Environmental Earth Sciences. 2016; 75(8):632 10.1007/s12665-016-5413-z

[38] Wu Z, Wu W, Lin C, Zhou S, Xiong J. Deciphering the origins, composition and microbial fate of dissolved organic matter in agro-urban headwater streams. Science of The Total Environment. 2019; 659:1484–1495. 10.1016/j.scitotenv.2018.12.237 31096358

[39] Arndt S, Jørgensen BB, Larowe DE, Middelburg JJ, Pancost RD, Regnier P. Quantifying the degradation of organic matter in marine sediments: A review and synthesis. Earth-Science Reviews. 2013; 123(4):53–86. 10.1016/j.earscirev.2013.02.008 10630702

[40] Jaiswal D, Pandey J. Anthropogenically enhanced sediment oxygen demand creates mosaic of oxygen deficient zones in the Ganga River: Implications for river health. Ecotoxicology and Environmental Safety. 2019; 171:709–720. 10.1016/j.ecoenv.2019.01.039 30658307

[41] Eyre BD, Ferguson AJ. Denitrification efficiency for defining critical loads of carbon in shallow coastal ecosystems. Hydrobiologia. 2009; 629(1):137–146. 10.1007/s10750-009-9765-1

[42] Zhu G, Shanyun W, Yixiao L, Zhuang L, Siyan Z, Cheng W, et al Microbial pathways for nitrogen loss in an upland soil. Environmental Microbiology. 2018; 20(5):1723–1738. 10.1111/1462-2920.14098 29528547

[43] Zhu G, Wang S, Zhou L, Wang Y, Zhao S, Xia C, et al Ubiquitous anaerobic ammonium oxidation in inland waters of China: an overlooked nitrous oxide mitigation process. Scientific Reports. 2015; 5(7):1256–1267. 10.1038/srep17306 26610807 PMC4661425

[44] Wang S, Wang W, Liu L, Zhuang L, Zhao S, Su Y, et al Microbial Nitrogen Cycle Hotspots in the Plant-Bed/Ditch System of a Constructed Wetland with N2O Mitigation. Environmental Science & Technology. 2018; 52(11):6226–6236. 10.1021/acs.est.7b04925 29750509

[45] Thamdrup B, Dalsgaard T. Production of N(2) through anaerobic ammonium oxidation coupled to nitrate reduction in marine sediments. Appl Environ Microbiol. 2002; 68(3):1312–1318. 10.1128/aem.68.3.1312-1318.2002 11872482 PMC123779

[46] Zhang L, Okabe S. Ecological niche differentiation among anammox bacteria. Water Research. 2020; 171:115468 10.1016/j.watres.2020.115468 31926373

[47] Dalsgaard T, Canfield DE, Petersen J, Thamdrup B, Acuna-Gonzalez J. N-2 production by the anammox reaction in the anoxic water column of Golfo Dulce, Costa Rica. Nature. 2003; 422(6932):606–608. 10.1038/nature01526 12686998

[48] Kuypers MMM, Sliekers AO, Lavik G, Schmid M, Jorgensen BB, Kuenen JG, et al Anaerobic ammonium oxidation by anammox bacteria in the Black Sea. Nature. 2003; 422(6932):608–611. 10.1038/nature01472 12686999

[49] Su X, Chen Y, Wang Y, Yang X, He Q. Impacts of chlorothalonil on denitrification and N2O emission in riparian sediments: Microbial metabolism mechanism. Water Research. 2019; 148:188–197. 10.1016/j.watres.2018.10.052 30388520

[50] Dalsgaard T, Stewart FJ, Thamdrup B, De Brabandere L, Revsbech NP, Ulloa O, et al Oxygen at nanomolar levels reversibly suppresses process rates and gene expression in anammox and denitrification in the oxygen minimum zone off northern Chile. mBio. 2014; 5(6):e01966 10.1128/mBio.01966-14 25352619 PMC4217175

[51] Sánchez-Carrillo S, Angeler DG, álvarez-Cobelas M, Rojo C. Abiotic drivers of consumer foodweb structure in lakes. Freshwater Science. 2018; 37(2):404–416. 10.1086/697927

[52] Logue JB, Stedmon CA, Kellerman AM, Nielsen NJ, Andersson AF, Laudon H, et al Experimental insights into the importance of aquatic bacterial community composition to the degradation of dissolved organic matter. The ISME Journal. 2016; 10(3):533–545. 10.1038/ismej.2015.131 26296065 PMC4817675

[53] Juliette LY, Hyman MR, Arp DJ. Inhibition of Ammonia Oxidation in Nitrosomonas europaea by Sulfur Compounds: Thioethers Are Oxidized to Sulfoxides by Ammonia Monooxygenase. Applied and Environmental Microbiology. 1993; 59(11):3718–3727. 10.1128/AEM.59.11.3718-3727.1993 16349086 PMC182523

[54] Delgado Vela J, Dick GJ, Love NG. Sulfide inhibition of nitrite oxidation in activated sludge depends on microbial community composition. Water Research. 2018; 138:241–249. 10.1016/j.watres.2018.03.047 29604576

[55] Straub KL, Benz M, Schink B, Widdel F. Anaerobic, nitrate-dependent microbial oxidation of ferrous iron. Applied and Environmental Microbiology. 1996; 62(4):1458–1460. 10.1128/AEM.62.4.1458-1460.1996 16535298 PMC1388836

[56] Zhang RC, Xu XJ, Chen C, Xing DF, Shao B, Liu WZ, et al Interactions of functional bacteria and their contributions to the performance in integrated autotrophic and heterotrophic denitrification. Water Research. 2018; 143:355–366. 10.1016/j.watres.2018.06.053 29986245

[57] Jaroszynski LW, Cicek N, Sparling R, Oleszkiewicz JA. Impact of free ammonia on anammox rates (anoxic ammonium oxidation) in a moving bed biofilm reactor. Chemosphere. 2012; 88(2):188–195. 10.1016/j.chemosphere.2012.02.085 22483855

[58] Strauss EA, Mitchell NL, Lamberti GA. Factors regulating nitrification in aquatic sediments: effects of organic carbon, nitrogen availability, and pH. Canadian Journal of Fisheries and Aquatic Sciences. 2002; 59(3):554–563. 10.1139/f02-032

[59] Lee SM, Jung JY, Chung YC. Measurement of ammonia inhibition of microbial activity in biological wastewater treatment process using dehydrogenase assay. Biotechnology Letters. 2000; 22(12):991–994. 10.1023/a:1005637203643

[60] Sonthiphand P, Cejudo E, Schiff SL, Neufeld JD. Wastewater Effluent Impacts Ammonia-Oxidizing Prokaryotes of the Grand River, Canada. Applied and Environmental Microbiology. 2013; 79(23):7454–7465. 10.1128/AEM.02202-13 24056472 PMC3837731

[61] Neufeld RD, Hill AJ, Adekoya DO. Phenol and free ammonia inhibition to Nitrosomonas activity. Water Research. 1980; 14(12):1695–1703. 10.1016/0043-1354(80)90105-0

[62] Laforest-Lapointe I, Paquette A, Messier C, Kembel SW. Leaf bacterial diversity mediates plant diversity and ecosystem function relationships. Nature. 2017; 546(7656):145–147. 10.1038/nature22399 28538736

[63] Maynard DS, Crowther TW, Bradford MA. Fungal interactions reduce carbon use efficiency. Ecology Letters. 2017; 20(8):1034–1042. 10.1111/ele.12801 28677157

[64] Chen L, Jiang Y, Liang C, Luo Y, Xu Q, Han C, et al Competitive interaction with keystone taxa induced negative priming under biochar amendments. Microbiome. 2019; 7(1):77 10.1186/s40168-019-0693-7 31109381 PMC6526607

