# Supplementary material for: Insight into the nitrogen accumulation in urban center river from functional genes and bacterial community
Source: PLoS One. 2020 Sep 2;15(9):e0238531. doi: 10.1371/journal.pone.0238531 (PMC7467313; doi:10.1371/journal.pone.0238531)
Supplement: S1 Fig — Linear regression analysis of the sediment TN (A) and TP (B) with the water. (DOCX) [file pone.0238531.s001.docx]

**S1 Fig. Linear regression analysis of the sediment TN (A) and TP (B) with the water.**
