# Supplementary material for: Insight into the nitrogen accumulation in urban center river from functional genes and bacterial community
Source: PLoS One. 2020 Sep 2;15(9):e0238531. doi: 10.1371/journal.pone.0238531 (PMC7467313; doi:10.1371/journal.pone.0238531)
Supplement: S1 Table — (DOCX) [file pone.0238531.s002.docx]

**S1 Table. Primers and programs of the target genes in the quantitative PCR analysis.**

| Target gene | Primers | Program |
| --- | --- | --- |
| 16S rRNA | 338F: ACTCCTACGGGAGGCAGCAG  518R: ATTACCGCGGCTGCTGG | Pre-denaturation at 95^o^C for 5 min, denaturation at 95^o^C for 15 s, annealing at 60^o^C for 1 min, and extension at 72^o^C for 1 min |
| *amoA* | amo598f: GAATATGTTCGCCTGATTG  amo718r: CAAAGTACCACCATACGCAG | Pre-denaturation at 95^o^C for 5 min, denaturation at 95^o^C for 15 s, annealing at 56^o^C for 45 s, and extension at 72^o^C for 30 s |
| *nxr* | F1norA: CAGACCGACGTGTGCGAAAG  R1norA: TCYACAAGGAACGGAAGGTC | Pre-denaturation at 95^o^C for 5 min, denaturation at 95^o^C for 15 s, annealing at 58^o^C for 30 s, and extension at 72^o^C for 30 s |
| *narG* | 1960m2f: TAYGTSGGGCAGGARAAACTG  2050m2r: CGTAGAAGAAGCTGGTGCTGTT | Pre-denaturation at 95^o^C for 5 min, denaturation at 95^o^C for 15 s, annealing at 58^o^C for 45 s, and extension at 72^o^C for 30 s |
| *napA* | V17F: TGGACVATGGGYTTYAAYC  4R: ACYTCRCGHGCVGTRCCRCA | Pre-denaturation at 95^o^C for 5 min, denaturation at 95^o^C for 15 s, annealing at 56^o^C for 45 s, and extension at 72^o^C for 30 s |
| *nirK* | 583F: TCATGGTGCTGCCGCGKGACGG  909R: GAACTTGCCGGTKGCCCAGAC | Pre-denaturation at 95^o^C for 5 min, denaturation at 95^o^C for 15 s, annealing at 64^o^C for 40 s, and extension at 72^o^C for 30 s |
| *nirS* | Cd3aF: AACGYSAAGGARACSGG  R3cd: GASTTCGGRTGSGTCTTSAYGAA | Pre-denaturation at 95^o^C for 5 min, denaturation at 95^o^C for 15 s, annealing at 57^o^C for 30 s, and extension at 72^o^C for 30 s |
| *norB* | 2F: GGNCAYCARGGNTAYGA  5R: ACCCANAGRTGNACNACCCACCA | Pre-denaturation at 95^o^C for 5 min, denaturation at 95^o^C for 15 s, annealing at 54^o^C for 30 s, and extension at 72^o^C for 30 s |
| *nosZ* | 1527F: CGCTGTTCHTCGACAGYCA  1773R: ATRTCGATCARCTGBTCGTT | Pre-denaturation at 95^o^C for 5 min, denaturation at 95^o^C for 15 s, annealing at 54^o^C for 50 s, and extension at 72^o^C for 30 s |
