# Supplementary material for: Insight into the nitrogen accumulation in urban center river from functional genes and bacterial community
Source: PLoS One. 2020 Sep 2;15(9):e0238531. doi: 10.1371/journal.pone.0238531 (PMC7467313; doi:10.1371/journal.pone.0238531)
Supplement: S2 Table — (DOCX) [file pone.0238531.s003.docx]

**S2 Table. The DOM component tryptophan to tyrosine ratio in FCS and LHS**

|  | FC-spring | FC-summer | LH-spring | LH-summer |
| --- | --- | --- | --- | --- |
| tryptophan / tyrosine | 5.58 | 6.57 | 3.02 | 1.64 |
|  | | | | |
