# Supplementary material for: Insight into the nitrogen accumulation in urban center river from functional genes and bacterial community
Source: PLoS One. 2020 Sep 2;15(9):e0238531. doi: 10.1371/journal.pone.0238531 (PMC7467313; doi:10.1371/journal.pone.0238531)
Supplement: S3 Table — (DOCX) [file pone.0238531.s004.docx]

|  | | pH | DO | TN | TOC | TP | NH_4_^+^-N | NO_3_^-^-N | SRP | α-diversity | 16SrDNA |  | AOB | *nxr* | *narG* | *napA* | *nirS* | *norB* | *nosZ* | denitrification rate |
| --- | --- | --- | --- | --- | --- | --- | --- | --- | --- | --- | --- | --- | --- | --- | --- | --- | --- | --- | --- | --- |
| DO | | 0.568 |  |  |  |  |  |  |  |  |  |  |  |  |  |  |  |  |  |  |
| TN | | -0.550 | -0.389 |  |  |  |  |  |  |  |  |  |  |  |  |  |  |  |  |  |
| TOC | | 0.571 | 0.356 | **-0.596*** |  |  |  |  |  |  |  |  |  |  |  |  |  |  |  |  |
| TP | | -0.480 | **-0.625*** | **0.860**** | -0.323 |  |  |  |  |  |  |  |  |  |  |  |  |  |  |  |
| NH_4_^+^-N | | -0.549 | **-0.963**** | 0.378 | -0.375 | **0.588*** |  |  |  |  |  |  |  |  |  |  |  |  |  |  |
| NO_3_^-^-N | | -0.212 | 0.404 | 0.552 | -0.429 | 0.257 | -0.379 |  |  |  |  |  |  |  |  |  |  |  |  |  |
| SRP | | **-0.592*** | -0.317 | 0.388 | **-0.624*** | 0.303 | 0.329 | 0.457 |  |  |  |  |  |  |  |  |  |  |  |  |
| α-diversity | | -0.174 | 0.389 | **0.584*** | -0.476 | 0.260 | -0.389 | **0.975**** | 0.437 |  |  |  |  |  |  |  |  |  |  |  |
| 16SrDNA | | -0.197 | **-0.753**** | -0.021 | 0.242 | 0.329 | **0.769**** | **-0.772**** | -0.195 | **-0.777**** |  |  |  |  |  |  |  |  |  |  |
| AOB | | **0.592*** | **0.918**** | -0.361 | 0.228 | **-0.613*** | **-0.937**** | 0.354 | -0.404 | 0.389 | **-0.776**** |  |  |  |  |  |  |  |  |  |
| *nxr* | | -0.218 | 0.193 | **0.753**** | -0.270 | 0.543 | -0.224 | **0.733**** | 0.081 | **0.777**** | -0.378 |  | 0.182 |  |  |  |  |  |  |  |
| *narG* | | -0.085 | -0.571 | -0.161 | 0.273 | 0.126 | **0.594*** | **-0.800**** | -0.241 | **-0.842**** | **0.860**** |  | **-0.650*** | -0.483 |  |  |  |  |  |  |
| *napA* | | **0.585*** | 0.186 | **-0.746**** | **0.690*** | -0.536 | -0.140 | **-0.744**** | **-0.807**** | **-0.777**** | 0.385 |  | 0.189 | **-0.608*** | 0.503 |  |  |  |  |  |
| *nirS* | | 0.099 | -0.487 | -0.445 | 0.399 | -0.123 | 0.434 | **-0.940**** | -0.386 | **-0.928**** | **0.797**** |  | -0.406 | **-0.650*** | **0.825**** | **0.636*** |  |  |  |  |
| *norB* | | **0.582*** | 0.214 | **-0.768**** | **0.860**** | -0.519 | -0.203 | **-0.754**** | **-0.750** | **-0.779**** | 0.417 |  | 0.151 | -0.564 | 0.522 | **0.939**** | **0.658*** |  |  |  |
| *nosZ* | | 0.413 | -0.007 | **-.673*** | 0.467 | -0.545 | -0.014 | **-0.823**** | -0.479 | **-0.786**** | 0.382 |  | 0.014 | **-0.749**** | 0.551 | **0.763**** | **0.664*** | **0.779**** |  |  |
| denitrification rate | | **0.721**** | 0.534 | **-0.763**** | **0.763**** | **-0.626*** | -0.453 | -0.319 | -0.504 | -0.358 | 0.025 |  | 0.425 | -0.365 | 0.063 | **0.670*** | 0.270 | **0.738**** | 0.333 |  |
| anammox rate | | 0.363 | 0.004 | -0.516 | 0.572 | -0.382 | 0.004 | **-0.736**** | **-0.628*** | **-0.714**** | 0.515 |  | -0.060 | -0.343 | 0.564 | **0.683*** | **0.641*** | **0.770**** | **0.588*** | 0.566 |
|  | *Correlation is significant at the 0.05 level (2-tailed) | | | | | | | | | | | | | | | | | | | |
|  | **Correlation is significant at the 0.01 level (2-tailed) | | | | | | | | | | | | | | | | | | | |

**S3 Table. Spearman's correlation coefficient of potential N removal rate with relative abundances of functional genes and environmental factors in overlying water.**
