# Supplementary material for: Insight into the nitrogen accumulation in urban center river from functional genes and bacterial community
Source: PLoS One. 2020 Sep 2;15(9):e0238531. doi: 10.1371/journal.pone.0238531 (PMC7467313; doi:10.1371/journal.pone.0238531)
Supplement: S4 Table — (DOCX) [file pone.0238531.s005.docx]

**S4 Table. Spearman's correlation coefficient of potential N removal rate with relative abundances of functional genes and environmental factors in sediment.**

|  | TN | TOC | TP | NH_4_^+^-N | NO_3_^-^-N | α-diversity | 16SrDNA | AOB | *nxr* | *narG* | *napA* | *nirS* | *nirK* | *norB* | *nosZ* | denitrification rate |
| --- | --- | --- | --- | --- | --- | --- | --- | --- | --- | --- | --- | --- | --- | --- | --- | --- |
| TOC | **0.701*** |  |  |  |  |  |  |  |  |  |  |  |  |  |  |  |
| TP | **0.916**** | **0.834**** |  |  |  |  |  |  |  |  |  |  |  |  |  |  |
| NH_4_^+^-N | 0.428 | 0.417 | 0.365 |  |  |  |  |  |  |  |  |  |  |  |  |  |
| NO_3_^-^-N | **0.620*** | **0.797**** | **0.676*** | **0.788**** |  |  |  |  |  |  |  |  |  |  |  |  |
| α-diversity | -0.119 | -0.345 | -0.314 | **0.584*** | 0.000 |  |  |  |  |  |  |  |  |  |  |  |
| 16SrDNA | -0.385 | **-0.713**** | -0.532 | **-0.627*** | **-0.846**** | 0.130 |  |  |  |  |  |  |  |  |  |  |
| AOB | -0.567 | **-0.734**** | **-0.602*** | **-0.739**** | **-0.937**** | 0.000 | **0.860**** |  |  |  |  |  |  |  |  |  |
| *nxr* | -0.301 | **-0.587*** | -0.357 | -0.389 | **-0.769**** | 0.389 | **0.776**** | **0.776**** |  |  |  |  |  |  |  |  |
| *narG* | -0.494 | **-0.727**** | **-0.592*** | **-0.757**** | **-0.916**** | 0.000 | **0.909**** | **0.958**** | **0.727**** |  |  |  |  |  |  |  |
| *napA* | -0.487 | **-0.741**** | **-0.578*** | **-0.746**** | **-0.909**** | 0.000 | **0.902**** | **0.944**** | **0.720**** | **0.993**** |  |  |  |  |  |  |
| *nirS* | **-0.581*** | **-0.713**** | **-0.630*** | **-0.739**** | **-0.958**** | 0.043 | **0.832**** | **0.965**** | **0.797**** | **0.944**** | **0.923**** |  |  |  |  |  |
| *nirK* | -0.571 | **-0.783**** | **-0.627*** | **-0.767**** | **-0.979**** | 0.000 | **0.818**** | **0.965**** | **0.769**** | **0.937**** | **0.930**** | **0.972**** |  |  |  |  |
| *norB* | -0.361 | -0.434 | -0.340 | **-0.984**** | **-0.776**** | **-0.583*** | **0.664*** | **0.727**** | 0.371 | **0.755**** | **0.741**** | **0.713**** | **0.748**** |  |  |  |
| *nosZ* | -0.551 | **-0.760**** | **-0.614*** | **-0.779**** | **-0.928**** | 0.000 | **0.928**** | **0.935**** | **0.760**** | **0.970**** | **0.963**** | **0.928**** | **0.918**** | **0.781**** |  |  |
| denitrification rate | -0.550 | **-0.811**** | **-0.606*** | **-0.725**** | **-0.958**** | 0.000 | **0.860**** | **0.944**** | **0.748**** | **0.916**** | **0.930**** | **0.916**** | **0.958**** | **0.727**** | **0.907**** |  |
| anammox rate | 0.147 | 0.140 | 0.301 | **-0.739**** | -0.378 | **-0.777**** | 0.259 | 0.413 | 0.217 | 0.378 | 0.371 | 0.392 | 0.406 | **0.734**** | 0.368 | 0.385 |
| *Correlation is significant at the 0.05 level (2-tailed) | | | | | | | | | | | | | | | | |
| **Correlation is significant at the 0.01 level (2-tailed) | | | | | | | | | | | | | | | | |
